# Can a short educational video improve knowledge of paediatric chronic pain among caregivers and healthcare professionals?

**DOI:** 10.1186/s13052-026-02376-6

**Published:** 2026-10-28

**Authors:** Annalisa Salerno, Francesca Rusalen, Vinci Cardona, Fulvia Salvi, Franca Benini

**Affiliations:** 1 Department of Women’s and Children’s Health, University of Padua, Padua, Italy; 2 Paediatric Pain and Palliative Care Service, Department of Women’s and Children’s Health, University Hospital of Padua, Padua, Italy; 3 MediCinema, Italia Onlus, Milan, Italy

**Keywords:** Paediatric chronic pain, Children, Educational video, Biopsychosocial model, Caregivers, Healthcare professionals

## Abstract

**Background:**

Chronic pain affects 20–46% of children, yet it remains underrecognized and undertreated, with significant consequences for daily functioning, school participation, and psychosocial well-being. Families play a central role in the biopsychosocial approach to management. Effective, accessible communication strategies may therefore represent valuable tools to improve knowledge of paediatric chronic pain and facilitate its recognition.

**Methods:**

We developed a short educational video on paediatric chronic pain, designed to deliver key information in a simple, engaging format suitable for both caregivers and healthcare professionals. To evaluate its impact, a structured questionnaire was administered to 288 participants (58% healthcare professionals, 42% caregivers/parents).

**Results:**

At baseline, healthcare professionals demonstrated higher knowledge (89% correct answers) compared with caregivers (59%). After viewing the video, caregivers showed statistically significant improvement in all 7 questions, nearly closing the initial gap. Among healthcare professionals, improvements were modest but consistent, reaching statistical significance in two questions. Overall satisfaction with the video was very high across both groups, who valued the clarity and relevance of the content.

**Conclusion:**

A short educational video was associated with improved immediate knowledge of paediatric chronic pain, especially among caregivers. Multimedia tools may represent a scalable complement to traditional interventions, supporting family empowerment and reinforcing the biopsychosocial approach. For healthcare professionals, however, these tools should be complemented by more tailored educational resources.

**Supplementary Information:**

The online version contains supplementary material available at https://doi.org/10.1186/s13052-026-02376-6.

## Background

Chronic pain in childhood is a prevalent but still underrecognized condition, with estimates suggesting that between 20% and 46% of children experience it in some form [1, 2]. The consequences extend far beyond the symptom itself: children affected by persistent pain often reduce or abandon school attendance, limit participation in sports and social activities, and may develop depressive symptoms, anxiety, or dependence on caregivers in daily functioning. The resulting burden impacts not only the child but also the entire family system, generating broader psychosocial consequences [3, 4].

For healthcare professionals, one of the greatest challenges is to adequately recognize, assess, and treat paediatric chronic pain. The longer pain persists without proper intervention, the more significant its effect on functional outcomes and psychosocial development. Effective management therefore requires a comprehensive, interdisciplinary approach in line with the biopsychosocial model, in which families play a central role [1, 5, 6].

Videos are widely used educational tools that can effectively communicate health information to broad audiences [7, 8]. Short multimedia resources may therefore enhance understanding of complex concepts across diverse populations [9].

The primary objective of this study was to evaluate whether a short educational video could improve healthcare professionals’ and caregivers’ knowledge of the correct identification and management of paediatric chronic pain. The secondary objective was to evaluate participants’ satisfaction with the educational video.

## Methods

This was a single-group pre-post interventional study conducted among healthcare professionals and caregivers. To achieve the primary objective, we created a 4-minute video, organised into five short chapters, providing key information on the recognition and management of paediatric chronic pain. The content was designed in a simple and empathetic manner to be easily understood by a heterogeneous audience including physicians, nurses, parents/caregivers, family members, and other individuals involved in children’s care.

The impact of the video on immediate knowledge acquisition was evaluated using a questionnaire developed by three physicians with expertise in paediatric chronic pain. The questionnaire addressed the same core concepts presented in the video and was developed based on the current literature. It was subsequently assessed by individuals without a healthcare background to ensure clarity and comprehensibility, although it did not undergo formal psychometric validation. It included demographic questions, seven multiple-choice knowledge questions addressing pain perception in neonates compared with adults, the role of acute pain, the definition and prevalence of chronic pain, its impact on health and quality of life, the importance of the biopsychosocial and multidisciplinary approach, and the role of multimodal (rather than exclusively pharmacological) treatment. Three additional multiple-choice questions evaluated participants’ satisfaction with the educational tool (clarity of the content, adequacy of the video’s duration, and quality of the graphic design).

Participants were recruited through an institutional dissemination campaign conducted at the Department of Women’s and Children’s Health, University of Padua, and among primary care paediatricians in the Padua area. Participants viewed the educational video in person on a tablet and completed the same online questionnaire immediately before and after viewing using the same device. Each correct answer received one point, whereas incorrect and “I don’t know” responses received zero points.

Pre-post differences in questionnaire responses were analysed using McNemar’s test for paired categorical data. Statistical significance was set at *p* < 0.05. Statistical analyses were performed using R software (version 4.3.3).

## Results

A total of 288 participants completed the questionnaire: 58% were healthcare professionals and 42% were caregivers/parents. Among professionals, 60% were physicians, of whom 70% were paediatric residents. Baseline knowledge was higher among healthcare professionals, who answered correctly on average 89% of the questions, compared with 59% among caregivers. After viewing the video, caregivers demonstrated a statistically significant improvement in all 7 questions (all *p* < 0.001), nearly closing the initial gap with professionals. The greatest improvement concerned the definition of chronic pain, with correct responses increasing from 17% before viewing the video to 96% afterwards (*p* < 0.001). Among healthcare professionals, improvements were more modest: the trend was positive across all questions, but statistical significance was reached only for the questions addressing the definition and prevalence of chronic pain (*p* < 0.001). Overall satisfaction with the video was very high in both groups, who reported strong appreciation for the clarity, accessibility, and relevance of the content. Detailed pre- and post-intervention results for each of the seven knowledge questions, stratified by participant group, are provided in Additional file 1.

## Discussion

Consistent with previous studies [7, 9, 10], our findings suggest that even brief exposure to clear, focused information on a specific topic — in this case, paediatric chronic pain — delivered through a simple video showed potential for improving immediate knowledge. However, improved knowledge should not be interpreted as evidence of behavioural change. Rather, it reflects participants’ immediate acquisition of the key concepts presented in the video. Importantly, this improvement was achieved without the support of a dedicated professional, underscoring the scalability of such tools as independent educational resources.

The greater improvement observed among caregivers compared with healthcare professionals was expected, as caregivers started from a lower baseline level of knowledge. In addition, the high baseline knowledge among healthcare professionals, together with the large proportion of paediatric residents included in the study, may have resulted in a ceiling effect, limiting the measurable improvement observed in this subgroup and the generalisability of these findings to more experienced clinicians and other healthcare disciplines. Because the educational content was intentionally simplified for a heterogeneous audience, healthcare professionals would likely benefit from more detailed and specialized resources.

Our findings should be interpreted in light of some limitations. The questionnaire was administered immediately after viewing the video; therefore, only immediate knowledge acquisition could be assessed. A potential testing or recall effect due to the repeated administration of the same questionnaire cannot be excluded. In addition, the absence of a control group prevents causal attribution of the improvement exclusively to the intervention, while the voluntary participation of both healthcare professionals and caregivers may have introduced some degree of selection bias. Although the questionnaire underwent expert content review and assessment of clarity among lay individuals, it did not undergo formal psychometric validation.

## Conclusions

Further studies are warranted to determine whether these improvements are retained over time and translate into meaningful changes in healthcare-seeking behaviour and clinical practice.

The video will be disseminated as part of the regional campaign “Diamo valore al dolore” (“Give value to pain”) across paediatric units, emergency departments, and primary care paediatricians’ offices in the Veneto region, with the aim of promoting earlier recognition and more appropriate management of paediatric chronic pain. Thanks to its universal visual language, it also lends itself to international adaptation through simple voice-over dubbing, offering opportunities for broader cross-cultural educational campaigns.

The video entitled Chronic Pain in Children – Educational Video from the Pediatric Hospice of Padova is freely available online at https://www.youtube.com/watch, with optional English subtitles. It can also be accessed by scanning the QR code below:

**Figure:**
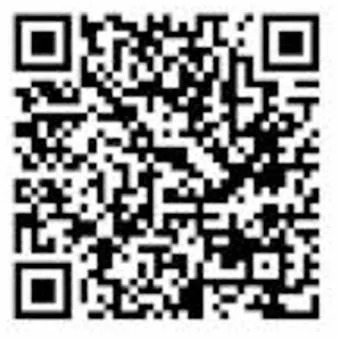

## Supplementary Information

Below is the link to the electronic supplementary material.

Supplementary Material 1

## Data Availability

The datasets generated and analysed during the current study are available from the corresponding author on reasonable request. The video is the property of the Paediatric Hospice of Padua and is freely available for educational and dissemination purposes. Any unauthorized reproduction is subject to legal action.

## References

[1] King S, Chambers CT, Huguet A, MacNevin RC, McGrath PJ, Parker L, MacDonald AJ. The epidemiology of chronic pain in children and adolescents revisited: A systematic review. Pain. 2011;152:2729–38. 10.1016/j.pain.2011.07.016. 22078064

[2] Mathews L. Pain in Children: Neglected, unaddressed and mismanaged. Indian J Palliat Care. 2011;17:70. 10.4103/0973-1075.76247. PMC3140088 21811376

[3] Palermo TM. Impact of Recurrent and Chronic Pain on Child and Family Daily Functioning: A Critical Review of the Literature. J Dev Behav Pediatr. 2000;21:58–69. 10.1097/00004703-200002000-00011. 10706352

[4] Gatchel RJ, McGeary DD, McGeary CA, Lippe B. Interdisciplinary chronic pain management: Past, present, and future. Am Psychol. 2014;69:119–30. 10.1037/a0035514. 24547798

[5] Dobe M, Zernikow B, editors. Practical treatment options for chronic pain in children and adolescents: an interdisciplinary therapy manual. Heidelberg; New York: Springer; 2013.

[6] Guidelines on the management of chronic pain in children. Geneva: World Health Organization; 2020. 33433967

[7] Zhu J, Yuan H, Zhang Q, Huang P-H, Wang Y, Duan S, Lei M, Lim EG, Song P. The impact of short videos on student performance in an online-flipped college engineering course. Humanit Soc Sci Commun. 2022;9:327. 10.1057/s41599-022-01355-6. PMC9510322 36187843

[8] Sweller J. Implications of cognitive load theory for multimedia learning. In: Mayer RE, editor. The Cambridge handbook of multimedia learning. 1st ed. Cambridge: Cambridge University Press; 2005. p. 19–30. 10.1017/CBO9780511816819.003.

[9] Savage I, Goodyer L. Providing information on metered dose inhaler technique: is multimedia as effective as print? Fam Pract. 2003;20:552–7. 10.1093/fampra/cmg510. 14507797

[10] Rosaasen N, Mainra R, Kukha-Bryson A, Nhin V, Trivedi P, Shoker A, Wilson J, Padmanabh R, Mansell H. Development of a patient-centered video series to improve education before kidney transplantation. Patient Educ Couns. 2018;101:1624–9. 10.1016/j.pec.2018.04.014. 29729857

