# Supplementary material for: Can a short educational video improve knowledge of paediatric chronic pain among caregivers and healthcare professionals?
Source: Ital J Pediatr. 2026 Oct 28;52:156. doi: 10.1186/s13052-026-02376-6 (PMC13718051; doi:10.1186/s13052-026-02376-6)
Supplement: Supplementary file 1 [file 13052_2026_2376_MOESM1_ESM.docx]

**Additional file 1: Correct responses before and after video viewing by participant group**

| **Knowledge question** | **Caregivers/parents (n = 121)** | | | **Healthcare professionals (n = 167)** | | |
| --- | --- | --- | --- | --- | --- | --- |
|  | **Before, n (%)** | **After, n (%)** | **p-value** | **Before, n (%)** | **After, n (%)** | **p-value** |
| Pain perception in neonates compared with adults | 78 (64%) | 115 (95%) | < 0.001 | 157 (94%) | 157 (94%) | 1.000 |
| Protective role of acute pain | 84 (69%) | 108 (89%) | < 0.001 | 153 (92%) | 158 (95%) | 0.302 |
| Definition of chronic pain (duration) | 21 (17%) | 116 (96%) | < 0.001 | 96 (57%) | 159 (95%) | < 0.001 |
| Prevalence of paediatric chronic pain | 48 (40%) | 118 (98%) | < 0.001 | 136 (81%) | 164 (98%) | < 0.001 |
| Importance of chronic pain assessment and treatment due to its impact on health and quality of life | 94 (78%) | 121 (100%) | < 0.001 | 164 (98%) | 167 (100%) | 0.248 |
| Importance of a multidisciplinary approach to chronic pain management | 94 (78%) | 120 (99%) | < 0.001 | 165 (99%) | 167 (100%) | 0.480 |
| Role of multimodal (rather than exclusively pharmacological) treatment | 77 (64%) | 117 (97%) | < 0.001 | 164 (98%) | 165 (99%) | 1.000 |

Data are presented as n (%). Pre- and post-intervention differences were assessed using McNemar's test for paired categorical data. Statistical significance was set at p < 0.05
